# Effects of Metal Oxides on Carbonation and Coking of High-Salinity Organic Wastewater

**DOI:** 10.1155/2020/6667497

**Published:** 2020-12-10

**Authors:** Jumei Ye, Zhuang Li, Chongcong Li, Tianya Li, Ziqiao Gao, Hui Dong

**Affiliations:** ^1^College of Petroleum Engineering, Liaoning Shihua University, Fushun, Liaoning 113001, China; ^2^School of Energy and Power Engineering, Dalian University of Technology, China; ^3^College of Chemistry and Chemical Engineering, Liaoning Normal University, China; ^4^China Liaohe Petroleum Engineering Co., Ltd.(LPE), China; ^5^SEPA Key Laboratory on Eco-Industry, Northeastern University, China

## Abstract

Slag is difficult to treat quantitatively due to the formation of a molten mixture in the carbonization process of high-salinity organic wastewater. Thus, aiming at solving this difficulty, the effects of metal oxide additives, additive ratio, furnace burden ratio, and carbonization temperature on the carbonization and coking of high-salinity organic wastewater are systematically analyzed. The analysis is performed using scanning electron microscopy, X-ray diffraction, and Vickers hardness tests. The results show that all five metal oxide additives can reduce the hardness of carbonized products. The relative effect of reducing the coked hardness is as follows: MgO > CaO > kaolin > Fe_2_O_3_ > Al_2_O_3_. Thus, the effect of MgO on reducing the coking hardness is stronger than that of the other four metal oxides, reducing the hardness of carbonized products by approximately 81%. Furthermore, the adding charge can reduce the hardness index by at least 60%. When the carbonization temperature is higher than 800°C, the hardness index of the carbonized product decreases by approximately 5% each 50°C of increase in temperature. This study shows that the addition of metal oxides can effectively reduce the hardness of coking during the treatment of high-salt organic wastewater by carbonization and oxidation and provide theoretical support for the subsequent treatment of high-salt organic wastewater by carbonization and oxidation.

## 1. Introduction

Domestic sewage, industrial wastewater, and other useless water, such as the inflow of the first rain path into the drainage pipes canals, are called wastewater. The direct discharge of wastewater can substantially damage the environment due to its complex composition. According to data from the China Environmental Statistics Yearbook, although the discharge of industrial wastewater in China showed a downward trend in the past decade, by 2018, the industrial wastewater discharge in China was still high, 18.16 billion tons, accounting for 23.55% of the national wastewater discharge. Industrial wastewater is characterized by having a wide range of sources, high salt concentrations, and complex components [1–3]. A report predicted that in 2025, China would produce more than 12 million tons [4] of recoverable resources from industrial wastewater, producing over 10 million tons of waste salt. The recovery and use of the waste salt from high-salinity organic wastewater, as industrial salt, represent a major source for reuse and circular economy. Therefore, the treatment of high-salinity organic wastewater is of paramount importance.

A large number of salts and other impurities are often present in hypersaline organic wastewater. When this water is treated in a high-temperature process such as incineration [5, 6], pyrolysis [7], and advanced oxidation processes [8], the water can cause problems such as high hardness index coking owing to adhesion between the salt and impurity in the organic wastewater. Currently, some treatment methods such as biological [9–12] and electrodialysis methods [13–15] allow treating the organic wastewater without requiring high temperatures. Moreover, these methods did not produce products with a high coking hardness index. However, these methods show limitations. The existing methods for treating hypersaline organic wastewater have their own advantages and disadvantages. During the process of incineration and pyrolysis, water in high-salinity organic wastewater is heated to evaporate and vaporize, separating saline water. Moreover, for the recovery of high-temperature flue gas generated in the treatment process, a method of adding an eternal magnetic field [16] or adopting a nanofluid medium [17] can be adopted to improve the waste heat recovery efficiency. The precipitation of soluble salts and the separation of heavy metals from hypersaline organic wastewater were performed using the incineration method proposed by Atanes et al. [18]. This is a simple and efficient process in wastewater treatment technology; thus, incineration is becoming a good alternative for industrialization. However, this method will cause problems such as material surface softening and equipment sticking due to uneven heating. Recently, Xie et al. [19] used the molten salt oxidation method to carbonize organic matter in waste liquid, separating the organic matter and salt separated carefully. The inherent ability of molten salt oxidation as an advanced oxidation technology is to destroy the organic components of waste while retaining inorganic components in the molten salts [20]. Lin et al. [21] showed that the waste liquid could be effectively oxidized in a molten salt bath. The temperature of molten carbonate plays a crucial role in the oxidizing process. When the temperature increased from 600°C to 750°C, the oxidation efficiency increased from 91.1% to 98.3%. Moreover, the influence of the air factor on the composition of the molten carbonate and the type of feed pipe was smaller than that of the temperature; the chlorine retention efficiency of molten carbonate was above 99.9%. They also found that when using molten salt oxidation of waste printed circuit boards [22], more than 95% of the copper was recycled, as the major component of ash, glass fiber is dissolved by molten carbonate and retained in the salt bath, obtaining an effective resource recovery.

Furthermore, biological methods have advantages such as low cost and wide application scope. In this regard, halophilic bacteria or salt-tolerant bacteria in sewage treatment processes could increase the salt content in wastewater or reduce the organic matter in wastewater. Some studies have shown that when the salt concentration is 10 mg/L, the COD removal rate becomes the highest [23]. However, if the salt concentration is very high or contains toxic components, the growth of microorganisms will be inhibited, affecting its treatment capacity and effectiveness. Wiśniewski [24] used an anion exchange membrane to remove bromate from waste salt during the Donnan dialysis process, and dechlorinated waste during the incineration process to reduce the damage to the environment of chloride ions [25]. The electrodialysis method can only remove acid radicals from high-salinity organic wastewater; however, it cannot achieve resource utilization.

In this study, the carbonization-oxidation method was used to treat organic wastewater with high salinity. According to the difference between the carbonization point of the organic substances in wastewater and the melting point of salt, the organic substances were carbonized. Subsequently, the carbonized product was dissolved to separate the carbon and salt. Unlike the molten salt oxidation method, the carbonization oxidation method can extract organic matter from wastewater and reuse it as an industrial raw material. Similar to fused salt oxidation, inorganic salts easily agglomerate with raw materials in the wastewater during carbonization and oxidation, resulting in partial loss of salt and a lower conversion rate of salt.

In conclusion, few studies have considered adding metal oxides to reduce the coking of carbonization products formed during the carbonization of high-salt organic waste liquid. In this study, five varieties of metal oxides (CaO, MgO, Fe_2_O_3_, Al_2_O_3_·2SiO_2_·H_2_O, and Al_2_O_3_) and shale ash (solid waste) were added to the furnace charge. The coking structures were analyzed under different conditions using X-ray diffraction (SEM) and scanning electron microscopy (XRD). The effects of CaO, MgO, Fe_2_O_3_, kaolin (Al_2_O_3_·2SiO_2_·H_2_O), and Al_2_O_3_ on the hardness index of the carbonized products and the effect of reducing the hardness of coke were also studied. The adequate additive and proportion, as well as the temperature, could be determined through the experiment. Therefore, this study can provide theoretical support for the subsequent carbonization-oxidation method to deal with high-salinity organic wastewater.

## 2. Materials and Methods

### 2.1. Materials

In this study, 1% toluene, 2% benzene, and 2% mixed xylene were added to a 10% NaCl solution to simulate high-salinity organic wastewater according to engineering specifications. Before the experiments, the quartz sand and shale ash were crushed to 40–60 mesh, washed with deionized water, and dried in a blast dryer at 110°C for 12 h.

In the experiment, the metal oxide additives were CaO, MgO, Fe_2_O_3_, kaolin, and Al_2_O_3_; their melting points are above 1500°C.  Table 1 shows the elemental analysis of the burden, considering the quartz sand and shale ash as the burden.

### 2.2. Equipment and Methods

The samples were heated in a muffle furnace (HY-1000MC) and dehydrated in a blast oven. Sodium chloride, furnace charge, and additives were weighed with a precise balance with an accuracy of 0.1 mg for accurate development of the experiment. An ultrasonic vibrator was used to thoroughly mix the sodium chloride, furnace charge, and additives. The hardness index (SII) of the carbonized products was measured using a Vickers hardness tester, and the coke after heating was tested by electron X-ray diffraction (XRD) and scanning electron microscopy (SEM) using a TD-3500 X-ray diffractometer and an electron microscope. The detailed experimental steps are shown in Table 2.

### 2.3. Identification Methods

The hardness of the carbonized product was evaluated using the hardness index SII. After the experiment, the hardness of the carbonized products was calculated using a Vickers hardness tester to develop a quantitative comparison. During the experiment, under the action of a particular load, an indentation with a square cone shape was created on the surface of the sample. The diagonal length of the indentation was measured to calculate the surface area of the indentation. The value of the load divided by the surface area represents the hardness of the sample. The calculation equation presented in [26] is written as follows:

(1) $$\begin{array}{c} HV=0.102\times \frac{2F\sin136/2}{d^{2}}\approx 0.1891\frac{F}{d^{2}}, \end{array}$$

where *F* is the test force (N),and *d* is the arithmetic mean of the diagonal lengths *d*_1_ and *d*_2_ of the two indentations (mm).

## 3. Results and Discussion

### 3.1. Effect and Mechanism of Metal Oxide Additives

**Figure 1:**
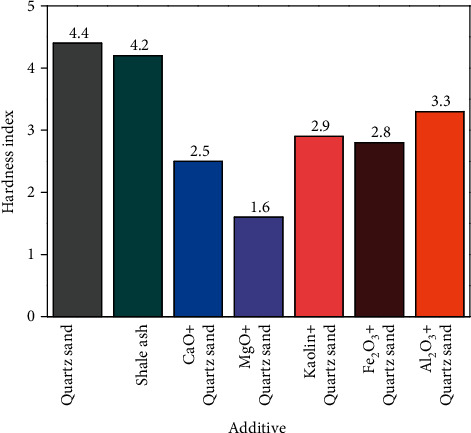
Effect of metal oxide additives on hardness index.

In each experiment, equal volumes of the developed hypersaline organic wastewater were added to different beakers. The quantity of the metal oxide additive was added to the beaker. The additive was added using a 1 : 1 molar mass ratio of the cation in the metal oxide to Na+ in the high-salt organic wastewater. The amount of the furnace charge was added to the beaker. The amount was added using a 1 : 1 mass ratio of the furnace charge to NaCl in the organic wastewater. After drying, the samples were heated in a corundum crucible, away from the door of the muffle furnace. After setting the temperature of the muffle furnace at 900°C for 30 min, the samples were carbonized after drying treatment.  Figure 1 shows the relationship between the hardness index, measured after carbonization, and the different types of additives.

The hardness index directly reflects the coking effect of carbonized products.  Figure 1 shows that when the same quality of shale ash and quartz sand is added into the two groups of high-salinity organic wastewater, and no metal oxide additives are added, the hardness index of the carbonated product is 4.2 and 4.4. Moreover, after adding CaO, MgO, Fe_2_O_3_, kaolin, or Al_2_O_3_, the hardness index of the high-salinity organic wastewater is reduced by at least 25%. Among them, the addition of MgO and CaO significantly reduced the hardness index of the product by 63.6% and 43.2%, respectively. The relative effects of the five metal oxides on the hardness index of carbonized products were Al_2_O_3_ < kaolin < Fe_2_O_3_ < CaO < MgO.

SEM and XRD enabled to observe the microstructure of the carbonized products to understand how metal oxide additives affect carbonized products of organic wastewater with high salinity. The specific analysis is discussed in the following paragraphs.

**Figure 2:**
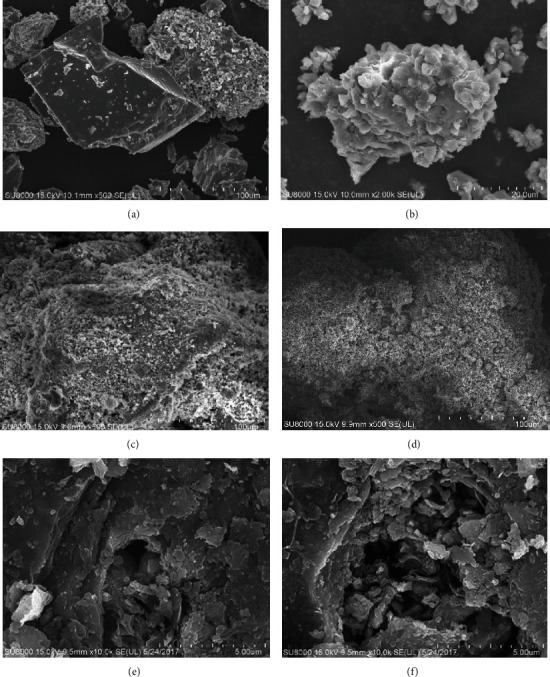
Microstructure of carbonization products. (a) No additions. (b) CaO. (c) MgO. (d) Fe_2_O_3_. (e) Kaolin. (f) Al_2_O_3_.

Figure 2 shows the microscopic morphology of the carbonized product of high-salt organic wastewater. In the absence of additives, the surface of the carbonized product is a polyhedral crystalline block, the surface is smooth without pores, and the edges and corners are clearly visible. In the experiment, no granular NaCl was present in the residual carbonization product. However, a crystalline substance was formed, which was attached to the bottom of the corundum crucible, caused by the mutual bonding of the quartz sand particles with the charge after melting [21]. After adding CaO, the edges and corners of the carbonized product vanished, and a large amount of particulate matter was formed on the surface. A small amount of molten NaCl was still present in the residual slag sample. Because CaO encapsulated the low-melting eutectic, a layer similar to a refractory material was formed on the surface of the residual slag sample. Moreover, the substance did not have cohesiveness; thus, it reduced the mutual adhesion of molten NaCl and furnace charge [22]. After adding MgO to the high-salinity organic wastewater, the surface of the carbonized product became rough to form several pores. Moreover, powdery substances were present on the surface, as well as more loose flocculent fillers between the carbonized products. The high melting point of MgO powder provided a dilution effect on the molten NaCl and formed a protective layer on the surface of the charged particles. In addition, the presence of flocculent fillers prevented the diffusion of molten NaCl and adsorbed more NaCl instead of further diffusing on the surface of the charged particles. After adding Fe_2_O_3_, the surface of the carbonized product becomes rough, but there is still obvious coking. After adding kaolin and Al_2_O_3_, the microscopic morphology of the carbonized products is similar. The surface of the charged particles was smooth, and the edges and corners were clear. Large gaps between the particles with no flocculent filler were observed. Moreover, less NaCl was present on the surface of the carbonized product in the molten state, which was because Al^3+^ and Si^4+^ are having similar ionic interaction forces with molten Na^+^, resulting in a similar degree of sintering on the particle surface [23].

**Figure 3:**
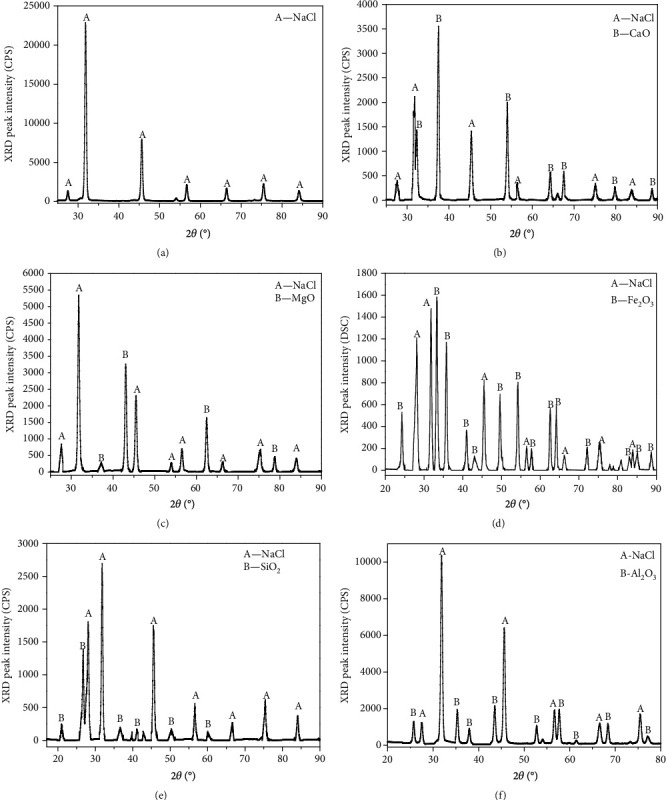
XRD analysis of carbonation products. (a) No additions. (b) CaO. (c) MgO. (d) Fe_2_O_3_. (e) Kaolin. (f) Al_2_O_3_.

The XRD analysis chart of the high-salinity organic-wastewater carbonized products is shown in Figure 3. No chemical reaction occurred in the process of heating and carbonization of the samples without any additional additives. Moreover, only NaCl was present in the carbonized products. Typically, sodium chloride and silicon oxide react as follows [27]:

(2) $$\begin{array}{c} 2\text{NaCl}+3\text{Si}{\text{O}}_{2}+{\text{H}}_{2}\text{O}=2\text{HCl}+\text{N}{\text{a}}_{2}\text{O}+3\text{Si}{\text{O}}_{2}. \end{array}$$

However, the chemical reaction between sodium chloride and silicon oxide did not occur because the sample was dried in a blast dryer before heating, and no additional water was added to the chemical reaction. This shows that only the physical reaction of NaCl occurred during the heating process. After adding CaO, the XRD analysis diagram of the carbonized products showed that only two crystal phases were present, CaO and NaCl, in the carbonized products, and no material with a high melting point was generated by the reaction between sodium silicate and CaO [28]. This again shows that the main mechanism of the coking of carbonized products is not a chemical reaction but a physical action. A high-melting-point CaO powder produces a dilution effect on molten NaCl, forming a protective layer on the surface of charged particles to prevent the molten NaCl powder from sticking to the particles to restrain coking. With the addition of MgO, only two crystalline phases of MgO and NaCl are present in the carbonized products, and the detectable intensity of NaCl clearly decreases. The flocculent filler formed by MgO on the surface of the carbonized products absorbed NaCl so that the amount of NaCl existing on the surface of the carbonated products substantially decreased. The XRD results of the carbonization products were similar after adding Fe_2_O_3_, kaolinite, or Al_2_O_3_, indicating that the three kinds of metal oxides had similar mechanisms to inhibit the coking of the carbonization products.

**Figure 4:**
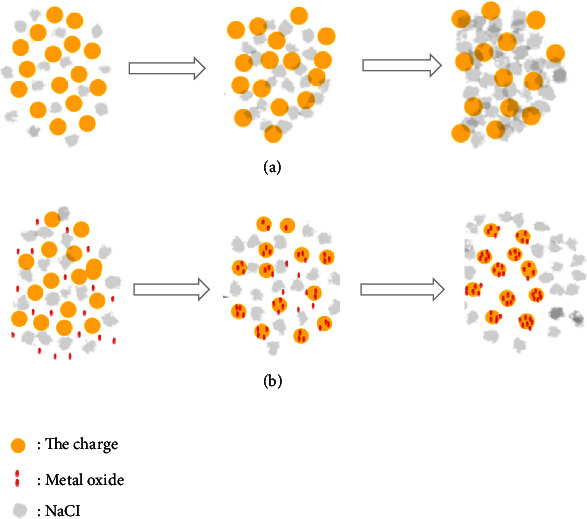
Schematic diagram of coking hardness reduction.

The results of SEM and XRD show that the metal oxide mechanism reduces the coking of the carbonized product, as shown in Figure 4. In particular, Figure 4(a) shows that when the high-salt organic wastewater is not carbonized with metal oxide after the furnace charge adheres to the surface of NaCl, the furnace charge adheres to each other. Moreover, the particles after the carbonization of organic matter adhere to each other, forming coking. As shown in Figure 4(b), the added additives adhere to the burden surface and form a protective layer; the organic matter carbonated particles and the burden are no longer bond with NaCl.

In this experiment, because the CaO wraps the low-melting-point eutectic, a layer of refractory-like material was formed on the surface of the residual slag sample, and the material was not cohesive, thus, reducing the bonding between the molten NaCl and the furnace charge [22]. After adding the MgO, the high-melting-point MgO powder could dilute the molten NaCl and formed a protective layer on the surface of the charged particles. Besides, the presence of flocculent filler could prevent the diffusion of molten NaCl better, and more NaCl was absorbed instead of further diffusing on the surface of the furnace charge particles. After adding Fe_2_O_3_, kaolin, and Al_2_O_3_, the microstructures of the carbonized products were similar. Moreover, a slightly molten state of NaCl remains on the surface of the carbonized products. This may be because the Fe^3+^ and Al^3+^ are positive trivalent cations, having similar interaction force with molten Na^+^, resulting in a similar sintering degree on the surface of the particles [23]. After Fe_2_O_3_, kaolin, and Al_2_O_3_ were added, the surfaces of the burden particles were smooth with clear edges and corners, with larger gaps between the particles. This indicates that the three additives may play a role in the framework for melting the ash melt, effectively raising the melting point of the eutectic to inhibit coking [29].

From the above results, owing to the protective effect of the furnace charge due to the additive, the NaCl adhered to the surface of the carbonized product was reduced, and more NaCl was separated; thus, the conversion rate of waste salt increased.

### 3.2. Effect of Additive Types

**Figure 5:**
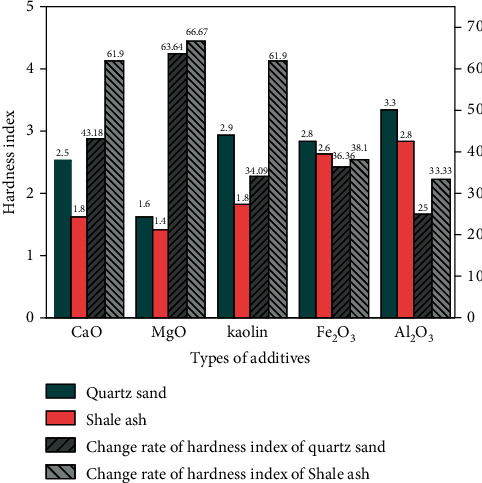
Effect of metal oxide additives on hardness index.

In the following experiment, the quartz sand considered in the previous section was replaced by shale ash with the same quality, and the remaining conditions remained unchanged. The hardness indexes of the carbonized products were measured after the experiment.  Figure 5 shows the effect of metal oxide additives on the hardness index.

Figure 4 shows that the hardness indexes of the carbonization products of the high-salt organic wastewater are 4.4 and 4.2 when the furnace charges are quartz sand and shale ash, respectively, without additional additives. As shown in Figure 5, the hardness index is reduced by at least 25% after adding the metal oxide additive. The hardness indexes can be reduced by 25–63.6% and 33.3–66.7% when using quartz sand and shale ash as the furnace charge, respectively. MgO shows a better effect on reducing the hardness index because the floccules produced by MgO absorb molten NaCl. Fe_2_O_3_ and Al_2_O_3_ have no significant effect on hardness reduction because Na^+^ in quartz sand interacts with Fe^3+^(Fe_2_O_3_) and Al^3+^ (Al_2_O_3_) [30]. Concerning the charging, the relative effect of the five additives on the hardness index of carbonized products was MgO > CaO > kaolin > Fe_2_O_3_ > Al_2_O_3_. In conclusion, MgO is an additive that can significantly reduce the coking caused by the cohesion of molten NaCl and furnace charge in processing high-salinity organic wastewater by carbonization-oxidation.

### 3.3. Effect of Additive Ratio

**Figure 6:**
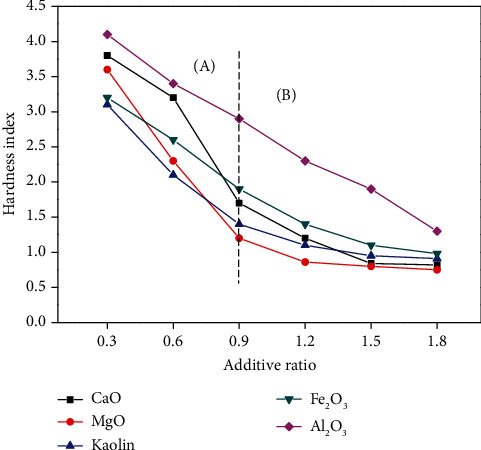
Effect of the metal oxide additives proportion on the hardness index.

Under the same conditions, different molar mass ratios of metal oxide additives (1 : 0.3, 1 : 0.6, 1 : 0.9, 1 : 1.2, and 1 : 1.8) were added to the organic wastewater with high salinity. The hardness indexes of the carbonized products were measured after being dried and carbonized. The relationship between the measured hardness index and the ratio of the additives is shown in Figure 6.

As shown in Figure 6, when the proportion of the metal oxide additive is 0.3, the hardness of the experimental residual slag decreases by 6.8–13.6%. The effect of the additive on reducing the hardness is not significant. The hardness decreases by 70.5–81.4% when the proportion of the additives is 1.8; thus, significantly reducing the coking. Moreover, Figure 6 can be divided into two regions, regions A and B, according to the change rate of the hardness index to the proportion of additives. In region A, the hardness index of the carbonized product reduces by approximately 20% on average for every 0.3 increments of the additive proportion. In contrast, in region B, with an increase in the additive proportion, the hardness index decrease is not evident. The hardness index of carbonized products is reduced by 6% when the additive proportion is increased every 0.3. The reason is that the crystal protective layer formed by the additives outside the sample particles created a certain hardness in the burden. When the proportion of additives increased gradually, the dilution effect of additives on NaCl was already large enough. No chemical reaction was present in the SEM analysis after adding additives, and the added additives could cover the furnace charge with a certain hardness. Thus, the rate of reducing the hardness index of each additive tends to be slow. Furthermore, as shown in Figure 6, the effect of preventing coking is always superior when using MgO as an additive. This result is similar to the microstructure analysis of the carbonized product because the flocculent produced by MgO adsorbs NaCl; thus, less NaCl adheres to the surface of the furnace charge than the other four additives [31].

### 3.4. Effect of Burden and Its Proportioning

**Figure 7:**
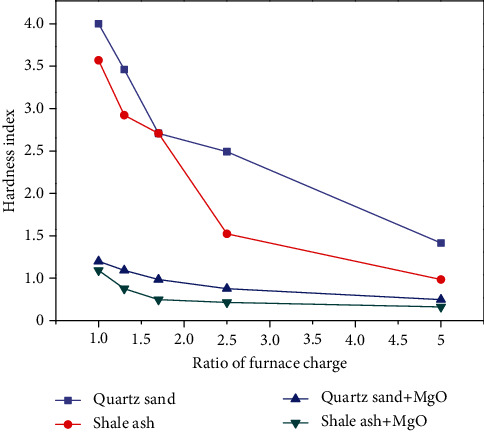
Effect of furnace charge and ratio on the hardness of carbonization products.

Three groups were considered based on adding different mass ratios of furnace charge (1 : 1, 1 : 1.3, 1 : 1.7, 1 : 2.5, and 1 : 5) to high-salinity organic wastewater. In the first group, no additional additive was added, and the furnace charge was shale ash. In the second group of experiments, the burden was shale ash without any additives. The third group was based on the first group of experiments, adding MgO, whose molar mass ratio is 1 : 1. The fourth group was based on the second group of experiments; however, the same proportion of MgO was added, in this case. After drying, the samples were placed in a muffle furnace, and the heating conditions were consistent with the above experiments. The relationship between the hardness index and the charge and its ratio is shown in Figure 7.

Figure 7 shows the linear relationship between the type and proportion of charge and the hardness index. As shown in the figure, the hardness index of the carbonation product decreases with an increase in the proportion of the furnace charge when no additive is considered. The type and proportion of charge and the hardness index can reduce the hardness index of the carbonation product by approximately 60%. When the burden proportion is less than 2.5, and the proportion of the burden increases for every 50%, the hardness index of the carbonation products decreases by 10% and 15% when the quartz sand and shale ash are used as the burden, respectively. When the burden proportion is above 2.5, and the proportion of the burden increases for every 50%, the hardness indexes of the carbonation products containing quartz sand and shale ash decrease by 4.5% and 2.3%, respectively. Thus, the hardness of carbonized products decreases with an increase in the burden ratio, showing that the burden hinders the NaCl melting-induced coking [32]. As the burden proportion continuously increases because of the hardness of the burden itself, the hardness index decreases with an increase in the burden proportion. Moreover, the effect of inhibiting coking caused by the further increase in the burden ratio is no longer evident. With the addition of MgO, the hardness index of carbide is reduced at least by 68.2%, which further verified the effect of additives on the hardness reduction of carbide. In addition, the effect of shale ash on hardness reduction is stronger than that of quartz sand under the same burden addition ratio, which is related to the composition of quartz sand and shale ash [30].

### 3.5. Effect of Temperature

**Figure 8:**
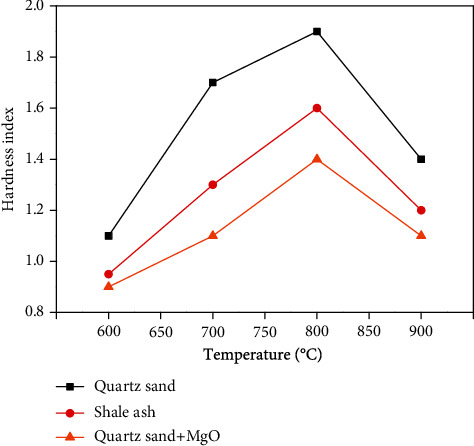
Effect of temperature on the hardness index.

The first group of samples used quartz sand as the furnace charge, while the second group of samples used shale ash as the furnace charge, without adding additional additives. In the third group of samples, quartz sand was used as the furnace charge, and MgO with a molar mass ratio of 1 : 1 was added. After drying the above three groups of samples, the hardness indexes of each sample at 600°C, 700°C, 800°C, and 900°C were measured, as shown in Figure 8.

Figure 8 shows that the hardness index of the carbonized products increases at first and subsequently decreases with an increase in temperature for the three experimental groups. The results show that when the temperature increases every 50°C, the hardness index increases by 4% until 800°C. In contrast, the hardness index decreases by approximately 5% when the temperature increases every 50°C for values higher than 800°C. The reason is that the melting temperature of NaCl is approximately 800°C. Therefore, when the temperature is below 800°C, the melting degree of NaCl increases with increasing temperature, thus, promoting the agglomeration of carbonized products and increasing its hardness. When the temperature is above 800°C, NaCl is in a molten state. With an increase in temperature, a small amount of NaCl may begin to evaporate on the surface of the carbonized product (in this process, the evaporated NaCl gas is not in molecular form), leading to an increase in the burden ratio and additive ratio. Thus, inhibiting the accumulation of carbonation products and reducing their hardness [29]. In the carbonization oxidation process of high-salt organic wastewater, the temperature can be set to above the melting point of salt to reduce the hardness of the coke.

## 4. Conclusions

The hardness index was introduced as an index to evaluate the hardness of carbonized products. The structure of the carbonized products after adding metal oxide additives was analyzed through SEM, XRD, and Vickers hardness test. The effects of the type and proportion of metal oxides, burden, and temperature on the hardness index of carbonized products were studied. The conclusions can be drawn as follows:

1. The relationship between the metal oxide additives and the hardness index of carbonized products showed that an increase in the molar mass ratio of the additives decreases the hardness index of the carbonized products significantly. The relative effect of the five metal oxide additives on reducing hardness was MgO > CaO > Kaolin > Fe_2_O_3_ > Al_2_O_3_. Among them, MgO can reduce the hardness index by approximately 80%.
2. Burden and temperature also affect the hardness index of carbonation products. The hardness index of the carbonization products decreases with an increase in the added burden ratio. When quartz sand and shale ash were used as the furnace charge, the hardness index decreased by approximately 60% and 66%, respectively. For temperatures above 800°C, the hardness index decreased by approximately 10% for every 50°C increments
3. Carbonization and oxidation technology can recycle the inorganic salts and organic matter in high-salt organic wastewater largely. This research can effectively solve the problem of coking in carbonization and oxidation treatment of wastewater. Therefore, adding metal oxide additives can increase the recovery rate of inorganic salts and organics in organic wastewater in actual industrial production. However, this study uses a laboratory to configure a mixed liquid to simulate high-salt organic wastewater and does not consider the impact of other particles in the actual wastewater. In future research, a wider range of metal oxide species should be selected, and the impact of other particles in actual industrial wastewater should be considered

## Figures and Tables

**Table 1:** Elemental analysis of quartz sand and shale ash.

Analyzed element	O	Na	Mg	Al	Si	P	Fe	Others
Quartz sand	55.9	0.03	-	0.19	43.81	-	0.03	0.04
Shale ash	45	-	1.42	12.28	27.13	1.27	7.19	5.71

Note: “-” indicates that no such element is present in the tested component.

**Table 2:** The experimental steps.

Order	Step
1	Number the beakers and add the charge and additives as required.
2	Adding an organic solution of sodium chloride prepared by using deionized water into a beaker.
3	The beaker was stirred thoroughly and placed in an ultrasonic vibrator for 2 hours.
4	After completely mixing the NaCl in the beaker, the furnace charge, and the additive, the mixture was dried in a blast dryer at a temperature of 120°C for 4 hours.
5	Lay the dried sample on the bottom of the corundum crucible with a thickness of more than 5 mm, blow the crucible with nitrogen, cover the crucible, and heat it in a muffle furnace far away from the box door. The temperature shall be subjected to the requirements of the experimental group.
6	After being heated for 30 minutes, the samples were extracted to conduct the hardness test or SEM, and the experimental data were recorded.
7	Repeat the experiment three times and to obtain the average value.

## Data Availability

The data used to support the findings of this study are available from the corresponding author upon request.

## References

[1] Malik S. N., Ghosh P. C., Vaidya A. N., Mudliar S. N. (2019). Ozone pre-treatment of molasses-based biomethanated distillery wastewater for enhanced bio-composting. *Journal of Environmental Management*.

[2] Ma W. W., Han Y. X., Xu C. Y. (2018). Biotoxicity assessment and toxicity mechanism on coal gasification wastewater (CGW): a comparative analysis of effluent from different treatment processes. *Science of The Total Environment*.

[3] Liu H. B., Wang H. N., Zhou X., Fan J. L., Liu Y. F., Yang Y. (2019). A comprehensive index for evaluating and enhancing effective wastewater treatment in two industrial parks in China. *Journal of Cleaner Production*.

[4] Wang Y., Mei X., Ma T. F. (2018). Green recovery of hazardous acetonitrile from high-salt chemical wastewater by pervaporation. *Journal of Cleaner Production*.

[5] Lee M. K., Kang S. (2019). A study of salt-assisted solution combustion synthesis of magnesium aluminate and sintering behaviour. *Ceramics International*.

[6] Li X. D., Lv J. H., Xu M. R., Ma J. Y., Yan J. H., Cen K. F. (2005). Agglomeration characteristics in fluidized bed incineration of organic-condensed wastewater. *Journal of Chemical Industry and Engineering (China)*.

[7] Lee Y. E., Jo J. H., Kim I. T., Yoo Y. S. (2017). Chemical characteristics and NaCl component behavior of biochar derived from the salty food waste by water flushing. *Energies*.

[8] Sun Y., Yu L. L., Huang H. B., Yang J. W., Cheng S. A. (2017). Research trend and practical development of advanced oxidation process on degradation of recalcitrant organic wastewater. *Journal of Chemical Industry and Engineering (China)*.

[9] Ebrahimi A., Kebria D. Y., Darzi G. N. (2018). Improving bioelectricity generation and COD removal of sewage sludge in microbial desalination cell. *Environmental Technology*.

[10] Nelson M. J., Nakhla G., Zhu J. (2017). Fluidized-bed bioreactor applications for biological wastewater treatment: a review of research and developments. *Engineering*.

[11] Zhuang H. F., Han H. J., Shan S. D., Xue X. D. (2016). Advanced treatment of coal chemical wastewater using a novel MBBR process with short-cut biological nitrogen removal. *Journal of Chemical Industry and Engineering (China)*.

[12] Hu W. Y., Zhou Y., Min X. (2018). The study of a pilot-scale aerobic/Fenton/anoxic/aerobic process system for the treatment of landfill leachate. *Environmental Technology*.

[13] Luiz A., McClure D. D., Lim K. (2017). Potential upgrading of bio-refinery streams by electrodialysis. *Desalination*.

[14] Pisarska B., Jaroszek H., Mikołajczak W. (2017). Application of electro-electrodialysis for processing of sodium sulphate waste solutions containing organic compounds: preliminary study. *Journal of Cleaner Production*.

[15] Le Han S. G., Balmann H. R.-d. (2016). Transfer of neutral organic solutes during desalination by electrodialysis: Influence of the salt composition. *Journal of Membrane Science*.

[16] Wang J., Li G., Zhu H., Luo J., Sundén B. (2019). Experimental investigation on convective heat transfer of ferrofluids inside a pipe under various magnet orientations. *International Journal of Heat and Mass Transfer*.

[17] Zheng D., Wang J., Chen Z. X., Baleta J., Sundén B. (2020). Performance analysis of a plate heat exchanger using various nanofluids. *International Journal of Heat and Mass Transfer*.

[18] Atanes E., Cuesta-Garcia B., Nieto-Marquez A., Fernandez-Martinez F. (2019). A mixed separation-immobilization method for soluble salts removal and stabilization of heavy metals in municipal solid waste incineration fly ash. *Journal of Environmental Management*.

[19] Xie K., Hu H., Cao J., Yang F., Yao H. (2019). A novel method for salts removal from municipal solid waste incineration fly ash through the molten salt thermal treatment. *Chemosphere*.

[20] Guggenheim T. L., Kloppenburg L. M., Poirier C. (2013). Purification and utilization of a formerly incinerated sodium nitrite bearing wastewater stream. *Green Processing and Synthesis*.

[21] Lin C., Chi Y., Jin Y. (2018). Molten salt oxidation of organic hazardous waste with high salt content. *Waste Management and Research*.

[22] Lin C., Yong C., Jin Y. (2017). Experimental study on treating waste printed circuit boards by molten salt oxidation. *Waste and Biomass Valorization*.

[23] Aslan S., Sekerdag N. (2015). Salt inhibition on anaerobic treatment of high salinity wastewater by upflow anaerobic sludge blanket (UASB) reactor. *Desalination and Water Treatment*.

[24] Wiśniewski J. A., Kabsch-Korbutowicz M., Łakomska S. (2014). Ion-exchange membrane processes for Br^−^ and BrO^3−^ ion removal from water and for recovery of salt from waste solution. *Desalination*.

[25] Dai S., Zheng Y., Zhao Y., Chen Y., Niu D. (2019). Molten hydroxide for detoxification of chlorine-containing waste: unraveling chlorine retention efficiency and chlorine salt enrichment. *Journal of Environmental Sciences*.

[26] Hervas I., Montagne A., Van Gorp A., Bentoumi M., Thuault A., Iost A. (2016). Fracture toughness of glasses and hydroxyapatite: a comparative study of 7 methods by using Vickers indenter. *Ceramics International*.

[27] Acharya P. (1997). Process challenges and evaluation of bed agglomeration in a circulating bed combustion system incinerating red water. *Environmental Progress*.

[28] Corsino S. F., Capodici M., Torregrossa M., Viviani G. (2018). A comprehensive comparison between halophilic granular and flocculent sludge in withstanding short and long-term salinity fluctuations. *Journal of Water Process Engineering*.

[29] Xu L. G., Huang Y. J., Wang J., Zou L., Yue J. F. (2018). High-temperature corrosion properties of water wall material 15CrMoG under reducing atmosphere. *Journal of ZheJiang University (Engineering Science)*.

[30] Mysen B., Virgo D. (1986). Volatiles in silicate melts at high pressure and temperature: 2. Water in melts along the join NaAlO. *Chemical Geology*.

[31] Li Y. S., Duan Y. F., Liu M., Li N., Chen C., Lv J. H. (2019). Reaction parameters and mechanism of flocculent multifunction polyphenylene sulfide filter. *China Environmental Science*.

[32] Mousa E., Senk D., Babich A. (2010). Reduction of pellets-nut coke mixture under simulating blast furnace conditions. *Steel Research International*.

